# The Bilateral Ovariectomy in a Female Animal Exacerbates the Pathogenesis of an Intracranial Aneurysm

**DOI:** 10.3390/brainsci10060335

**Published:** 2020-05-31

**Authors:** Mieko Oka, Isao Ono, Kampei Shimizu, Mika Kushamae, Haruka Miyata, Takakazu Kawamata, Tomohiro Aoki

**Affiliations:** 1 Department of Molecular Pharmacology, Research Institute, National Cerebral and Cardiovascular Center, Osaka 564-8565, Japan; happy.harmony.toto@gmail.com (M.O.); onoisao@kuhp.kyoto-u.ac.jp (I.O.); k.shimizu.830923@gmail.com (K.S.); marojiji9@yahoo.co.jp (M.K.); hmiyata27@gmail.com (H.M.); 2 Core Research for Evolutional Science and Technology (CREST) from Japan Agency for Medical Research and Development (AMED), National Cerebral and Cardiovascular Center, Osaka 564-8565, Japan; 3 Department of Neurosurgery, Tokyo Women’s Medical University, Tokyo 162-8666, Japan; tkawamata@twmu.ac.jp; 4 Department of Neurosurgery, Kyoto University Graduate School of Medicine, Tokyo 606-8507, Japan; 5 Department of Neurosurgery, Showa University, Tokyo 142-8666, Japan; 6 Department of Neurosurgery, Shiga University of Medical Science, Shiga 520-2192, Japan

**Keywords:** intracranial aneurysm, subarachnoid hemorrhage, estrogen, female, endothelial cell, macrophage

## Abstract

Considering the poor outcome of subarachnoid hemorrhage (SAH) due to the rupture of intracranial aneurysms (IA), mechanisms underlying the pathogenesis of IAs, especially the rupture of lesions, should be clarified. In the present study, a rat model of IAs in which induced lesions spontaneously ruptured resulting in SAH was used. In this model, the combination of the female sex and the bilateral ovariectomy increased the incidence of SAH, similar to epidemiological evidence in human cases. Importantly, unruptured IA lesions induced in female animals with bilateral ovariectomy were histopathologically similar to ruptured ones in the presence of vasa vasorum and the accumulation of abundant inflammatory cells, suggesting the exacerbation of the disease. The post-stenotic dilatation of the carotid artery was disturbed by the bilateral ovariectomy in female rats, which was restored by hormone replacement therapy. The in vivo study thus suggested the protective effect of estrogen from the ovary on endothelial cells loaded by wall shear stress. β-estradiol or dihydrotestosterone also suppressed the lipopolysaccharide-induced expression of pro-inflammatory genes in cultured macrophages and neutrophils. The results of the present study have thus provided new insights about the process regulating the progression of the disease.

## 1. Introduction

Considering the devastating outcome of subarachnoid hemorrhage (SAH) due to the rupture of an intracranial aneurysm (IA) [1,2], the development of a novel therapeutic strategy to prevent the rupture of IAs is mandatory for social health. Mechanisms underlying the rupture of lesions should, therefore, be clarified. Thus, we attempted to find a cue from well-established epidemiological evidence. Epidemiological studies have consistently demonstrated a higher incidence of SAH in older females or postmenopausal females [3,4,5,6,7,8]. Based on the necessity of clarifying underlying mechanisms of the rupture of IAs, we examined whether sex difference is indeed present, and if present, why, using the already-established animal model of IAs [9].

## 2. Materials and Methods

### 2.1. IA Models of Rats and Histological Analysis of Induced IA

All of the following experiments, including animal care and use, complied with the National Institute of Health’s Guide for the Care and Use of Laboratory Animals and complied with the National Institute of Health’s Guide for the Care and Use of Laboratory Animals and were approved by the Institutional Animal Care and Use Committee of the National Cerebral and Cardiovascular Center (Approved number; #18010 and #19036). The present manuscript also adheres to the ARRIVE (Animal Research: Reporting of In Vivo Experiments) guidelines for reporting animal experiments.

Ten-week-old male or female Sprague−Dawley (SD) rats were purchased from Japan SLC (Slc:SD, Shizuoka, Japan) (*n* = 62 in total). Animals were maintained on a 12-h light/dark cycle, and had free access to feed and water. To induce IAs, the rats were subjected to ligation of the left carotid artery, the right external carotid artery and the right pterygopalatine artery, and systemic hypertension by the combination of a high salt diet and the ligation of the left renal artery under general anesthesia by the intraperitoneal injection of pentobarbital sodium (50 mg/kg, Somnopentyl, Kyoritsuseiyaku Corporation, Tokyo, Japan) and the inhalation of Isoflurane (1.5%–2%, #IYESC-0001, Pfizer Inc., New York, NY). In some female rats, the bilateral ovariectomy was also applied [9]. Immediately after the above surgical manipulations, animals were fed chow containing 8% sodium chloride and 0.12% 3-aminopropionitrile (#A0408, Tokyo Chemical Industry, Tokyo, Japan), an irreversible inhibitor of lysyl oxidase catalyzing the cross-linking of collagen and elastin. Animals that died within one week after the above surgical manipulations were excluded from the analyses. At 16 weeks after the surgical manipulations, blood pressure was measured by a tail-cuff method without any anesthesia and was calculated as an average of three measurements. Animals were then deeply anesthetized by an intraperitoneal injection of pentobarbital sodium (200 mg/kg), and transcardially perfused with 4% paraformaldehyde solution. The circle of Willis was then stripped from the brain surface and an IA lesion induced at the anterior communicating artery or the posterior communicating artery was dissected as a ruptured or unruptured lesion, according to the macroscopic observation of whether the clot or hemosiderin deposition was present around IA lesions. Here, ruptured IAs were exclusively induced at these sites examined in the model [9]. All the dead animals after at least one week of surgical manipulations were autopsied to examine the onset of SAH due to rupture of induced IAs. Histopathological examination was done after Elastica van Gieson, which visualizes the internal elastic lamina using 5-um-thick frozen sections. The size of induced lesions was analyzed using the slices with the maximum area selected from serial sections using ImageJ software (https://imagej.nih.gov/ij/index.html).

### 2.2. Immunohistochemistry

At the indicated period after the aneurysm induction, 5-µm-thick frozen sections were prepared. After blocking with 3% donkey serum (#AB_2337258, Jackson ImmunoResearch, Baltimore, MD, USA), slices were incubated with primary antibodies, followed by incubation with secondary antibodies conjugated with a fluorescence dye (Jackson ImmunoResearch). Finally, fluorescent images were acquired using a confocal fluorescence microscope system (FV1000 or FV3000, Olympus, Tokyo, Japan).

The following primary antibodies were used: mouse monoclonal anti-CD68 antibody (#ab31630, Abcam, Cambridge, UK), rabbit polyclonal anti-myeloperoxidase (MPO) antibody (#ab9535, Abcam), rabbit polyclonal anti-tumor necrosis factor (TNF)-alpha antibody (#ab6671, Abcam), mouse monoclonal anti-smooth muscle α-actin (SMA) antibody (#M0851, Dako, Agilent, Santa Clara, CA, USA).

The following secondary antibodies were used; Alexa Fluor 488-conjugated donkey anti-mouse IgG H&L antibody (#A21202, Thermo Fisher Scientific, Waltham, MA, USA), Alexa Fluor 488-conjugated donkey anti-rabbit IgG H&L antibody (#A21206, Thermo Fisher Scientific), Alexa Fluor 594-conjugated donkey anti-mouse IgG H&L antibody (#A21203, Thermo Fisher Scientific).

### 2.3. Stenosis Model of the Carotid Artery of a Rat

Female rats underwent a bilateral ovariectomy and sham operation, and were then maintained for 7 days before subjecting to the model. The left common carotid artery of rats was then ligated using a 10-0 nylon thread with 25 gauge needle put on the side of the artery and stenosis was established by removing only the needle [10,11]. The post-stenotic dilatation of the carotid artery was observed for 30 min after ligation.

### 2.4. Hormone Replacement Therapy

Estradiol valerate (1 mg/kg, #224136400 Pelanin Depot, Mochida Pharmaceutical Co., Ltd., Tokyo, Japan) was intramuscularly injected every 7 days in a female rat that underwent the bilateral ovariectomy.

### 2.5. Cell Line and Culture

RAW264.7 cell line (#TIB-71), used as a macrophage, and HL-60 cell line (#CCL-240), used as a neutrophil, were purchased from ATCC (Manassas, VA, USA) and maintained in Dulbecco’s Modified Eagle’s Medium (DMEM) (#044-32955, FUJIFILM Wako Pure Chemical Corporation, Osaka, Japan) supplemented with 10% or 20% fetal bovine serum (#FB-1365/500, Biosera, Nuaille, France), respectively.

### 2.6. Quantitative Real Time (RT)-PCR Analysis in Cultured Cells

RAW264.7 cells or HL-60 cells were pre-treated with β-estradiol (E2, 50 µg/mL, #E0025, Tokyo Chemical Industry) or 5α-Dihydrotestosterone (DHT, 50 µg/mL, #A0462, Tokyo Chemical Industry) for 24 h or 3 h, respectively. Cells were then stimulated with vehicle (Veh), LPS (1 µg/mL, #L2654, Sigma Aldrich, St. Louis, MO, USA) or TNF-α (100 ng/mL, R&D SYSTEMS, Minneapolis, MN, USA) for additional 60 min.

Total RNA was purified from stimulated cells and reverse-transcribed using a RNeasy Mini Kit (#74106, QIAGEN, Hilden, Germany) and a High-capacity cDNA Reverse Transcription Kit (#4368813, Life Technologies Corporation, Carlsbad, CA, USA), according to the manufacturers’ instructions. For quantification of gene expression, quantitative RT-PCR was performed on a LightCycler 480 (Roche, Indianapolis, IN, USA) with a TB Green Premix Ex Taq II (#RR820, TAKARA BIO INC., Shiga, Japan). Expression of *Actb* (a gene coding β-actin) in experiments using the RAW264.7 cell line, or *ACTB* in experiments using the HL-60 cell line, were used as internal controls. For quantitation, the second derivative maximum method was used for determining the crossing point.

Primer sets used are listed as follows: forward 5′-CACCTCAGGGAAGAATCTGG-3′ and reverse 5′-CATTCCTGAGTTCTGCAAAGG-3′ for *Tnf*; forward 5′-AAAGGGAGCTCCTTAACATGC-3′ and reverse 5′-CTTCCTGGGAAACAACAGTGG-3′ for *Il1b*; forward 5′-AATGATGTGTACGGCTTCAGG-3′ and reverse 5′-CTGTACAAGCAGTGGCAAAGG-3′ for *Ptgs2* (a gene encoding cyclooxygenase-2 (COX-2)); forward 5′-GCACAGACCTCTCTCTTGAGC-3′ and reverse 5′-ACCTGCTGCTGCTACTCATTCACC-3′ for *Ccl2* (a gene encoding monocyte chemoattractant protein-1 (MCP-1)); 5′-ACGACCAGAGGCATACAGGGA-3′ and 5′-CCCTAAGGCCAACCGTGAAA-3′ for *Actb*; forward 5′-TCAGCAATGAGTGACAGTTGG-3′ and reverse 5′-ATAGGCTGTTCCCATGTAGCC-3′ for *TNF*; forward 5′-CAAGCTGGAATTTGAGTCTGC-3′ and reverse 5′-ATTCAGCACAGGACTCTCTGG-3′ for *IL1B*; forward 5′-ACACCCTCTATCACTGGCATCC-3′ and reverse 5′-AACATTCCTACCACCAGCAACC-3′ for *PTGS2*; forward 5′-AGCTTCTTTGGGACACTTGC-3′ and reverse 5′-ATAGCAGCCACCTTCATTCC-3′ for *CCL2* and forward 5′-CATACTCCTGCTTGCTGATCC-3′ and reverse 5′-GATGCAGAAGGAGATCACTGC-3′ for *ACTB.*

### 2.7. Statistical Analysis

Data are shown as the mean ± SEM. Statistical comparisons between two or more groups were conducted using a Welch’s *t*-test or the Tukey-Kramer method, respectively, with JMP Pro 14 (SAS Institute Inc., Cary, NC, USA). A *p* value less than 0.05 was defined as statistically significant.

## 3. Results

### 3.1. Highest Incidence of SAH in Female Rats with the Bilateral Ovariectomy

In reference to established epidemiological evidence, the risk of SAH is higher in postmenopausal or older females than in males and in females before menopause [3,4,5,6,7,8], we first examined whether the sex difference in the incidence of SAH could be reproduced or not in a rat model of IAs [9] as in human cases, to escape many uncontrollable confounding factors. Rats were subjected to an IA model and the incidence of IA lesions at the anterior or the posterior communicating artery complex and the onset of SAH due to rupture of IA lesions at each site were examined.

**Figure 1:**
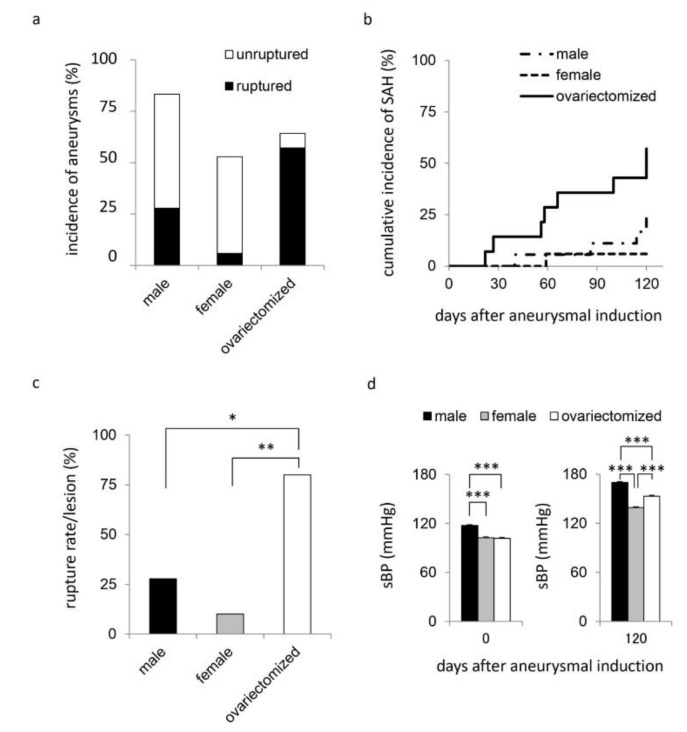
The incidence of intracranial aneurysms, the cumulative incidence of subarachnoid hemorrhage and the rate of rupture of induced lesions. To induce intracranial aneurysms, 10-week-old male (*n* = 18) or female Sprague−Dawley rats (*n* = 31) were subjected to the ligation of the left carotid artery, the right external carotid artery and the right pterygopalatine artery, and systemic hypertension by the combination of a high salt diet and the ligation of the left renal artery. In some female rats (*n* = 14), the bilateral ovariectomy was also applied. Animals were maintained for 120 days after surgical manipulations. The incidence of intracranial aneurysms (**a**), the cumulative incidence of subarachnoid hemorrhage (SAH) (**b**), the rate of rupture of induced lesions (**c**) or systolic blood pressure (sBP) (**d**) in each group; male rats, female rats without ovariectomy (female) or female rats with the bilateral ovariectomy (ovariectomized), are shown. Bars in (**d**) indicate the mean ± SEM. Statistical analysis was done by the Tukey-Kramer method in (**c**,**d**). * *p* < 0.05, ** *p* < 0.01, *** *p* < 0.001.

In male rats, 15 among 18 animals (83.3%) developed IAs at the anterior or the posterior communicating artery and 3 of these animals had multiple lesions at both artery complex. SAH occurred in 5 among 18 rats or IA lesions (27.8%) during the observation period of 16 weeks after the induction (Figure 1a–c). In female rats without the bilateral ovariectomy, 9 among 17 animals (52.9%) developed IAs at the anterior or the posterior communicating artery and one of these animals had multiple lesions. SAH occurred in 1 among 17 rats (5.9%) or 10 lesions (10.0%) in total in this group (Figure 1a–c). In female rats with bilateral ovariectomy, 9 among 14 animals (64.3%) developed IAs at the anterior or the posterior communicating artery and 1 of these animals had multiple lesions. SAH occurred in 8 among 14 rats (57.1%) or 10 lesions (80.0%) in total in this group (Figure 1a–c). Although the difference in sex or the implementation of ovariectomy did not influence the development of IAs, the combination of these two factors significantly facilitated the rupture of induced IAs in a rat model. The incidence of SAH per induced IA lesions was thus the highest in female rats with bilateral ovariectomy in spite of the significantly lower systolic blood pressure in female rats than in male rats (Figure 1).

### 3.2. Exacerbation of the IA Pathology in Female Rats with the Bilateral Ovariectomy

**Figure 2:**
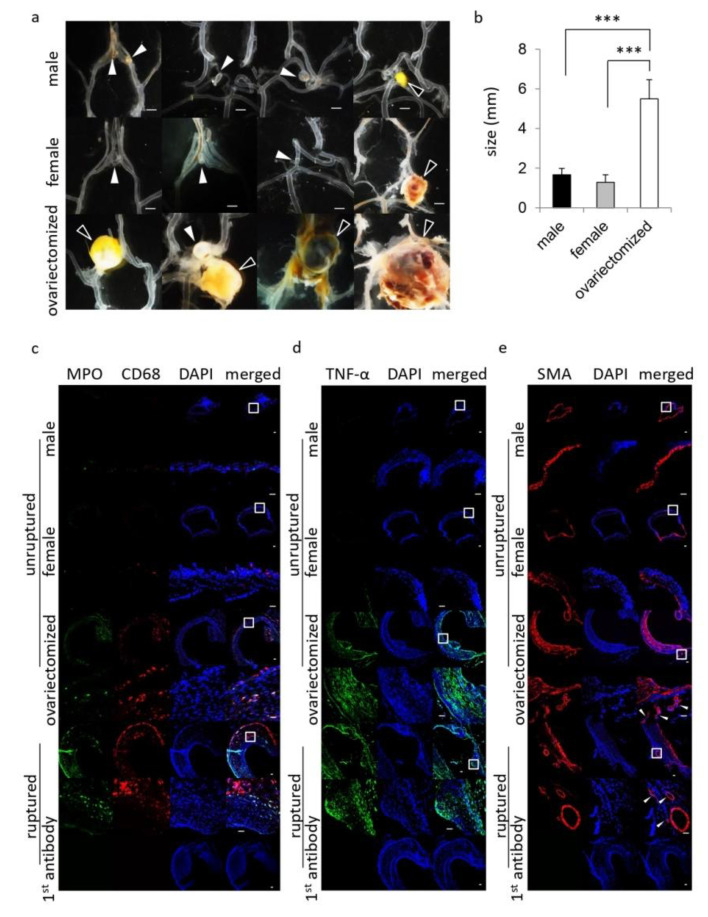
The exacerbation of the pathology of intracranial aneurysm in female rats with the bilateral ovariectomy. Ten-week-old female Sprague−Dawley rats were subjected to the aneurysm model. At 120 days after the surgical manipulations, specimens of induced lesions were harvested. (**a**,**b**) The macroscopic image of induced lesions (**a**) and their size (**b**) in each group; male rats (*n* = 18), female rats without ovariectomy (female, *n* = 10) or female rats with the bilateral ovariectomy (ovariectomized, *n* = 10). Bar, 1.0 mm (**a**). The white arrows and the black ones in (**a**) indicate the unruptured and the ruptured lesions, respectively. Bars in (**b**) indicate the mean ± SEM. Statistical analysis was done by the Tukey–Kramer method. ***; *p* < 0.001. (**c**,**d**) show similarity of unruptured lesions induced in female animals with the bilateral ovariectomy with ruptured ones. The representative images of immunostaining for a marker for neutrophil, myeloperoxidase (MPO, green in (**c**)), a marker for macrophage, CD68 (red in (**c**)), TNF-α (green in (**d**)), a marker for smooth muscle cell, smooth muscle alpha-actin (SMA, red in (**e**)), nuclear staining by DAPI (blue) or merged images are shown. The immunostaining without a 1st antibody served as a negative control and the representative images of this staining are shown in the lowest panels. Ruptured lesions were from female rats with the bilateral ovariectomy. The arrow in (**e**) indicates vasa vasorum with SMA-positive media. The magnified images, corresponding to a square in the upper panels, are shown in the lower panels. Bar, 20 μm.

To explore mechanisms underlying the effect of sex difference or the implementation of ovariectomy in female animals on the onset of SAH, the histopathological examinations of induced IA lesions were done. The size of induced IAs was significantly larger in female rats with bilateral ovariectomy than that in other groups (Figure 2a,b). Most IA lesions induced in female animals with bilateral ovariectomy ruptured, resulting in SAH (Figure 1). Intriguingly, the remaining unruptured lesions induced were apparently bigger than the ones induced in female animals without the ovariectomy or in male animals (Figure 2a). Immunohistochemical analyses revealed the accumulation of MPO-positive neutrophils and CD68-positive macrophages in arterial walls of the lesions, even in unruptured IA lesions specifically from female animals with bilateral ovariectomy, similarly to ruptured ones (Figure 2c). Such an accumulation of inflammatory cells was only limited in unruptured IA lesions from male animals or female animals without ovariectomy (Figure 2c). Consistently, expression of pro-inflammatory factor TNF-α, which is related with the pathogenesis [12,13,14], was higher in unruptured lesions from female animals with bilateral ovariectomy than that in male animals or female animals without ovariectomy (Figure 2d). In addition, expression of TNF-α in unruptured lesions from female animals with bilateral ovariectomy was similar to that in ruptured lesions (Figure 2d). In addition, the presence of vasa vasorum with SMA-positive media, which we identified as a histopathological characteristic of ruptured IA lesions [9], could be detected even in unruptured lesions only from female animals with the bilateral ovariectomy, and not in female animals without ovariectomy or male animals (Figure 2e). The unruptured IA lesion induced in female animals with the bilateral ovariectomy thus resembles ruptured lesions. In other words, the bilateral ovariectomy in female animals promotes events underlying rupture of the lesions.

### 3.3. Disturbance in Endothelial Function by the Bilateral Ovariectomy in Female Rats

**Figure 3:**
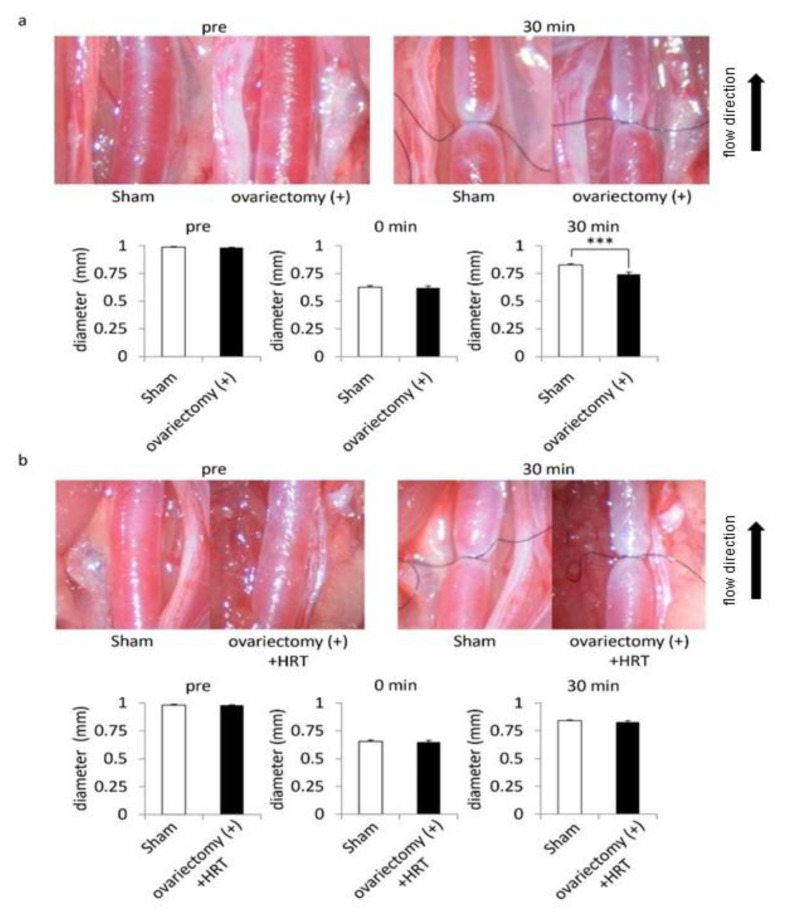
The disturbance of post-stenotic dilatation by the bilateral ovariectomy in female rats. 10-week-old female Sprague−Dawley rats were subjected to the bilateral ovariectomy or a sham-operation. On the 7th day, animals underwent the carotid ligation and the post-stenotic dilatation was observed for following 30 min (**a**). In some animals, hormone replacement therapy (HRT) was applied after the bilateral ovariectomy (**b**). Representative macroscopic images of the carotid artery before (pre) and 30 min after the ligation (30 min) are shown. The diameter of the carotid artery was calculated before (pre), just after (0 min) and 30 min after the ligation (30 min). Bars indicate the mean ± SEM (*n* = 4). Statistical analysis was done by a Welch’s *t* test. ***; *p* < 0.001.

A series of studies about IAs has clarified the involvement of macrophage-mediated chronic inflammatory responses in the pathogenesis of the disease and also the potential contribution of wall shear stress to this process [15,16,17,18,19,20]. To explore mechanisms regulating ovariectomy-mediated facilitation of rupture of lesions, we examined the effect of the bilateral ovariectomy on endothelial cell function by using the stenosis model of the carotid artery in which the post-stenotic dilatation occurs in response to increased wall shear stress-loading [10,11]. In female rats without ovariectomy, the post-stenotic dilatation of the carotid artery could be observed at 30 min after the partial ligation, as expected (Figure 3a). However, in female animals with the bilateral ovariectomy, the post-stenotic dilatation was partially but significantly restricted (Figure 3a). Importantly, hormone replacement therapy by Estradiol valerate restored ovariectomy-induced restriction of post-stenotic dilatation (Figure 3b). The bilateral ovariectomy thus disturbed endothelial cell function.

### 3.4. Suppressive Effect of Sex Hormone on Inflammatory Responses in Macrophages and Neutrophils

**Figure 4:**
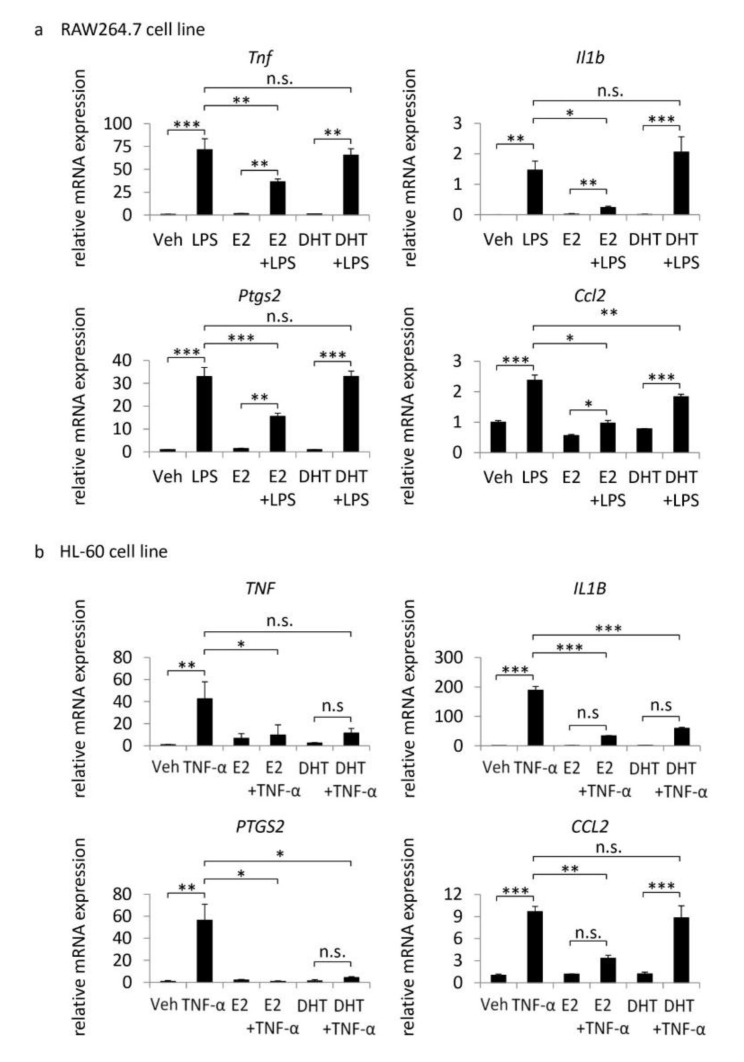
Suppressive effect of the sex hormone on expressions of pro-inflammatory genes in cultured macrophages or neutrophils. RAW264.7 cells (**a**) or HL-60 cells (**b**) were pre-treated with β-estradiol (E2, 50 µg/mL) or 5α-dihydrotestosterone (DHT, 50 µg/mL) for 24 h or 3 h, respectively. Cells were then stimulated with vehicle (Veh), LPS (dose) or TNF-α (dose) for an additional 60 min. Expressions of pro-inflammatory genes were examined by quantitative RT-PCR analyses. Bars indicate the mean ± SEM (*n* = 4). Statistical analysis was done by a Tukey-Kramer method. *; *p* < 0.05, **; *p* < 0.01, ***; *p* < 0.001. n.s.; statistically not significant.

Further, we examined the effect of the sex hormone, E2 (the hormone from the ovary) or DHT (the hormone from the testis), on inflammatory responses by cultured macrophages (RAW264.7 cell line) or neutrophils (HL-60 cell line). In RAW264.7 cells, although LPS-induced expressions of pro-inflammatory genes are related with pathogenesis, Tnf (TNF-α) [12,13,14], Il1b (IL-1β) [21], Ptgs2 (COX-2) [22] or Ccl2 (MCP-1) [20,23]—even with the pre-treatment by E2, the addition of E2 could significantly suppress expressions of all of these genes compared with those in the vehicle-treated cells (Figure 4a). The pre-treatment with DHT significantly suppressed expression of Ccl2 among four genes examined (Figure 4a). In HL-60 cells, the pre-treatment of E2 or DHT suppressed LPS-induced expression of pro-inflammatory genes (Figure 4b) in RAW264.7 cells. The suppressive effect of E2 was stronger than that of DHT as well (Figure 4b). Consistently, expression of TNF-α was higher in lesions from female animals with bilateral ovariectomy than that in male animals or female animals without ovariectomy (Figure 2d). The results of the in vitro study suggest the suppressive effect of the sex hormone on the inflammatory responses in lesions promotes the pathology.

## 4. Discussion

The epidemiological findings that post-menopausal females have a higher incidence of IAs than do males or females with menopause [3,4,5,6,7,8] was reproduced in the rat model in the present study, in which the bilateral ovariectomy in female animals significantly increased rupture of IAs. In another animal model, in which SAH was induced by the combination of the bilateral ovariectomy in female animals with the intrathecal injection of elastase, hormone replacement therapy by estrogen was shown to ameliorate the incidence of rupture [24]. The different animal models of SAH have thus consistently demonstrated the promoting effect of the bilateral ovariectomy on the rupture of IAs, confirming the crucial contribution of estrogen to the rupture of IAs. The previous experimental studies using an animal model of IAs have also demonstrated the facilitation of the formation and the progression of IAs by bilateral ovariectomy, which could be ameliorated by hormone replacement therapy [25,26]. In this report, similar to the present study, the protective role of estrogen in endothelial cell function has been indicated [26]. Furthermore, in human cases, the protective effect of hormone replacement therapy to compensate for the defect in functions of the ovary on the onset of SAH was reported [4,27], suggesting the clinical relevance of the present study. Hormone replacement therapy has adverse effects, such as the increased risk of breast cancer, ischemic stroke and ischemic heart disease, which makes the application of this therapy for the treatment of IAs in post-menopausal women controversial. However, the present study has provided experimental evidence for the potential of hormone replacement therapy as an option of treatment to prevent the onset of SAH in post-menopausal women.

Recent experimental studies mainly using an animal model of IAs [28,29] have clarified the involvement of chronic inflammatory responses in the process regulating the initiation, progression or rupture of IAs [15,16,18,30,31]. Additionally, hemodynamic force, especially wall shear stress, is considered a mediator of IA formation and progression, mainly through a series of studies by computational fluid dynamics analyses [17,32]. In the present study, we clarified the suppressive effect of E2 or DHT on expressions of pro-inflammatory factors in cultured macrophages and neutrophils (Figure 4). Additionally, the combination of female sex with bilateral ovariectomy exacerbated inflammatory cells like macrophages or neutrophils in lesions (Figure 2). Here, the in vitro finding that the suppressive effect of E2 was stronger than that of DHT may be responsible for the highest incidence of rupture in female animals with bilateral ovariectomy. Furthermore, in the stenosis model, the bilateral ovariectomy in female animals disturbed the high wall shear stress-induced post-stenotic dilatation of the carotid artery (Figure 3), suggesting the malfunction of endothelial cells. Intriguingly, the results of the present study have implied the role of the maladaptation of endothelial cells to shear stress-loading at the bifurcation sites as a trigger of molecular events, leading to the progression and rupture of the lesions. The ovariectomy in female animals, therefore, facilitates the pathogenesis of IAs in multiple steps by influencing the functions of endothelial cells and inflammatory cells.

## 5. Conclusions

To explore mechanisms regulating rupture of IAs, we have used a rat model and revealed the facilitation of the progression or rupture of the lesions by the combination of the female sex and the bilateral ovariectomy. Furthermore, we have clarified the point of actions of sex hormone as endothelial cells and inflammatory cells to inhibit the progression of the pathogenesis. The results of the present study have thus provided new insights about mechanisms regulating the progression of the disease.
